# Health State Monitoring of Bladed Machinery with Crack Growth Detection in BFG Power Plant Using an Active Frequency Shift Spectral Correction Method

**DOI:** 10.3390/ma10080925

**Published:** 2017-08-09

**Authors:** Weifang Sun, Bin Yao, Yuchao He, Binqiang Chen, Nianyin Zeng, Wangpeng He

**Affiliations:** 1 Institute of Intelligent Equipment and Smart Manufacturing, School of Aerospace Engineering, Xiamen University, Xiamen 361005, China; Vincent_suen@126.com (W.S.); yaobin@xmu.edu.cn (B.Y.); heyuchao1993@126.com (Y.H.); zny@xmu.edu.cn (N.Z.); 2 School of Aerospace Science and Technology, Xi’an 710075, China; hewp@xidian.edu.cn

**Keywords:** structural health monitoring, crack detection, power plant, blast furnace gas, centrifugal compressor, bladed machinery, spectral correction

## Abstract

Power generation using waste-gas is an effective and green way to reduce the emission of the harmful blast furnace gas (BFG) in pig-iron producing industry. Condition monitoring of mechanical structures in the BFG power plant is of vital importance to guarantee their safety and efficient operations. In this paper, we describe the detection of crack growth of bladed machinery in the BFG power plant via vibration measurement combined with an enhanced spectral correction technique. This technique enables high-precision identification of amplitude, frequency, and phase information (the harmonic information) belonging to deterministic harmonic components within the vibration signals. Rather than deriving all harmonic information using neighboring spectral bins in the fast Fourier transform spectrum, this proposed active frequency shift spectral correction method makes use of some interpolated Fourier spectral bins and has a better noise-resisting capacity. We demonstrate that the identified harmonic information via the proposed method is of suppressed numerical error when the same level of noises is presented in the vibration signal, even in comparison with a Hanning-window-based correction method. With the proposed method, we investigated vibration signals collected from a centrifugal compressor. Spectral information of harmonic tones, related to the fundamental working frequency of the centrifugal compressor, is corrected. The extracted spectral information indicates the ongoing development of an impeller blade crack that occurred in the centrifugal compressor. This method proves to be a promising alternative to identify blade cracks at early stages.

## 1. Introduction

The iron and steel manufacturing industry is one of the most energy-intensive industries in the world. As a kind of byproduct, blast furnace gas (BFG) is an important type of waste gas generated during blast furnace operations where iron ore is reduced with coke into metallic (pig) iron [1]. It is reported that approximately 1500~2000 m^3^ of BFG can be emitted on condition that one ton of pig iron is produced. Containing combustible and contaminating gases such as CO and CH_4_, the emission of BFG without proper processing emerges as a major source of the fog and haze in China. However, owing to its property of low heat value, it is relatively difficult to realize repeat utilization of BFG. In current steel-making operations, the development of furnace gas utilization for power generation is rapidly promoted because it enables alternative disposal of the problem gas while simultaneously harnessing it as an energy source [2].

The system of a BFG power plant consists of multiple types of rotary mechanical components such as motors, bearings, gears, and other bladed machinery. Normal and safe operation of these mechanical systems is of vital importance to ensure highly efficient reutilization of BFG. However, these mechanical components usually operate at severe working conditions of high temperature, varying working speed, and heavy load. Therefore, failures due to fatigue are likely to occur, resulting in not only economic loss but also catastrophic accidents [3]. To prevent major mechanical downtime, effective online condition monitoring techniques, which can be applied to the BFG power generation plant, are indispensable. In recent decades, researchers have paid attention to developments in these techniques [4,5,6]. Currently, health state monitoring and fault diagnosis of motors, bearings, and gears have received extensive research [7,8,9]. A considerable amount of attention is focused on the hot topic of advanced signal processing approaches for the analysis of vibration signals collected from the above rotary machinery [10,11,12]. The evolution of such advanced signal processing techniques with important improvements is beneficial for the understanding and interpretation of complex dynamic behaviors of machineries. Such techniques include wavelet transform [13,14], empirical mode decomposition [15,16], sparse representations [17,18], as well as time-frequency analyzing approaches [19,20].

Compared with other rotary components (bearings, gears, and motors), in situ dynamic analysis of bladed machinery is more difficult. Among the various failure types of bladed machinery, a significant problem that has not been properly addressed is the detection of crack growth. Doing work to the input BFG via impeller blades, the bladed machinery can generate high-pressure output gases. Because the working impeller blades must be exposed to extreme environments of high temperatures and corrosive BFG at high speeds, cracks are inevitable after long service. However, it is extremely difficult to detect incipient cracks using online condition monitoring techniques, especially at early stages of crack development. Typically, a fatigue crack becomes conspicuous only after the crack reaches about 80% of the total fatigue life of a structure [21]. This problem has attracted research interest from the scientists and engineers. At present, there are various non-destructive evaluation and structural health monitoring techniques that are available to detect the occurrence of cracks in laboratory environments. These techniques incorporate = ultrasonic, acoustic emission, thermal imaging, eddy current, magnetic particle inspection, X-ray, etc. measurements [22,23,24,25]. Among the various alternatives, a traditional contact measurement method is to mount strain gauges on the blade surface and record the testing signals using a data acquisition system [26,27,28]. Despite its effectiveness, this method is not developed such that it can overcome the hurdles to satisfy the needs of online testing. On the other hand, in state-of-the-art research, a considerable amount of attention has been paid to the analysis and identification of dynamic/modal parameters such as natural frequencies, mode shapes, and modal damping ratio from vibration signals of blade machinery for the purpose of crack detection.

In this paper, we attempted to monitor the health state of the bladed machinery in a BFG power plant. Vibration features, induced by a blade crack developed on a centrifugal compressor, were extracted using a novel signal processing approach. This approach derives harmonic information of high precision from the vibration signals. The varying of the health state of rotary machines can lead to a change in deterministic harmonic components, which are located in the lower frequency range of the collected dynamic signals. Traditionally, fast Fourier transform (FFT) is suitable for extracting information about these harmonic components. However, owing to the analyzing characteristics of FFT, severe distortions on information of frequency, amplitude, as well as phase are likely to occur [29]. In the past decades, a spectrum correction method has been developed for the purpose of high-precision correction of such information. Among these available techniques, ratio-based spectral correction methods, using FFT bins windowed by specific window functions, are popular with researchers due to their fast implementation and high precision. A large amount of new studies try to make theoretical contributions regarding the use of new types of windows [30,31,32,33]. Although some published articles have made comparisons in terms of the performance of these spectral correction techniques, very little attention has focused on the problem of retrieving precise harmonic information in the presence of strong corrupting noise, which is actually very common in practical applications.

To achieve good spectral correction performance in low signal-to-noise (SNR) conditions, some comparatively classical methodologies turn out to be very effective. These methodologies include zero-padded discrete Fourier transform (DFT) [34], the chirp Z-transform [35], and the Goertzel algorithm [36]. Zero-padded discrete DFT can be implemented via the algorithm of FFT, and it generated interpolated spectral bins across the entire frequency domain, therefore deriving information with high precision. However, the resulting accuracy is determined by the number of padded zeros. In some applications, the padded zeros may be hundreds or thousands of times that of the original signal in length. Chirp Z transform is similar to zero-padded DFT in its mathematical principle. Similarly, chirp Z transform can also approximate the continuous spectrum via interpolated spectral bins in a specific spectral range of interest. Chirp Z transform can be very time-consuming because computations of discretized spectral interpolation on the unit-circle in the Z plane must rely on the original DFT algorithm. The Goertzel algorithm is very efficient in calculating information of signals of single tone harmonic component. However, the precise frequency information should be available as prior knowledge for the Goertzel algorithm; otherwise it still demands a lengthy calculation period. To improve the efficiency of the above conventional algorithms, we propose an enhanced spectral correction method.

The power generation system studied in this paper is a celebrated power plant that had the highest burning rate (1131 km^3^/h) in the world in 2011. An accidental event occurred due to a fully developed blade crack on the centrifugal compressor, which is the key part of a booster fan. In the described case study, a range of vibration sensors were mounted on bearing housings of the shaft, on which the centrifugal compressor is installed such that vibration signals were collected. Ten vibration tests were conducted and a few records of velocity signals were collected via a data acquisition system. To investigate the harmonic information of the harmonic tones related to the fundamental working frequency, we propose a novel spectral correction technique with a post-processing step of active frequency shift operations on the FFT spectrum. Essentially, this proposed technique utilizes a conventional ratio-based spectral correction method on the information of interpolated Fourier spectral bins. The enhanced performance of the proposed active frequency-shift spectral correction (AFSSC) method is validated via numerical simulations. Sinusoidal signals corrupted with white Gaussian noise are employed to compare the performance of the proposed AFSSC with three other comparison methods. It is demonstrated that the proposed AFSSC using rectangular window possesses the most superior spectral correction capability. Moreover, a normalized health state indicator is further constructed using the corrected information to measure the energy weight of the harmonic tones. After using the health indicator, the energy weight of the component 2×, the second-order harmonic tone of the fundamental working frequency, successfully reveals the ongoing development of the blade crack.

## 2. Fundamentals of Ratio-Based Spectrum Correction

Fourier transform aims at decomposing a continuous signal into the sum of a few harmonic tones of various frequencies, shown as

(1) $$x(t)=\sum_{i=1}^{n}A_{i}\cos(2\pi f_{i}t+\phi_{i})+\varepsilon (t),$$

where ε(t) denotes the non-interesting interferences and the parameters $A_{i},f_{i},\phi_{i}$ denote the amplitude, frequency, and phase of a specific harmonic component, respectively. Conventional spectral correction techniques are based on discrete fast Fourier transform, which realizes fast approximations of continuous Fourier spectra. For a record of discrete digital signal x(n) with the sample length of L, its fast Fourier transform can be expressed as

(2) $$F{\left\{x\right\}}_{k}:=\sum_{n=0}^{L-1}x(n)\cdot \exp(-j\frac{2\pi kn}{L})\;,\;(k\in \mathbb{Z},\;j:=\sqrt{-1}).$$

From Equation (2), it is demonstrated that FFT only provides frequency information at the angular frequency w=2πk/L. Equivalently, the signal spectrum is discretized at evenly spaced points with a uniform resolution of 2π/L. Indexes of the discretized frequency bins are referred to as Fourier grids of FFT. As a result, precision harmonic information (amplitude, frequency, and phase) characterizing the sinusoidal components may be not directly available in the resulting spectrum. This is because, in discrete spectrum analysis, the picket-fence effect and the spectral aliasing effect are commonly unavoidable due to the limited duration observation interval and the difficulty of integral period truncation when sampling. As for those components without exact positive periodic sampling, errors occur in the spectral triad of {amplitude (*A*), frequency (*f*), and phase (ϕ)}. On the other hand, the presence of massive spectral bins, emerging in the form of nontrivial side lobes of an actual sinusoidal component, also complicates the extraction of exact information about harmonic components. To illustrate this problem, we use the following simple cosine signal of unique tone:

(3) $$x(t)=A\;\cos(2\pi f_{c}t+\phi ),$$

where the actual frequency of the existent harmonic component is $f_{c}$. Its Fourier transform can be represented as

(4) $$X(f)=\frac{A}{2}e^{j\phi }\delta (f-f_{c})+\frac{A}{2}e^{-j\phi }\delta (f+f_{c}).$$

In Equation (4), δ(t) is the Dirac delta function, which is defined as 1 at t=0 and zero at other t of nonzero values.

In actual measurements, x(t) is digitized using a time-shifted window $w_{T}(t)$ with a time length of T. Let the Fourier transform of $w_{T}(t)$ be denoted as

(5) $$W_{T}(f)=ℱ\{w_{T}(t)\}=|W_{T}(f)|e^{j\varphi (f)},$$

such that the corresponding Fourier transform of the windowed signal $w_{T}(t)x(t)$ can be expressed as $X(f)*W_{T}(f)$ on the basis of Fourier’s convolution theorem. If we concentrate on the positive frequency part of $w_{T}(t)x(t)$, the following expression can be obtained:

(6) $$X(f)*W_{T}(f)\approx \frac{A}{2}e^{j\phi }\delta (f-f_{c})*|W_{T}(f)|e^{j\varphi (f)}=\frac{A}{2}|W_{T}(f-f_{c})|e^{j[\varphi (f-f_{c})+\phi ]}.$$

In a specific realization of discrete Fourier transform, if the recorded sample length is L and the sampling frequency is $f_{s}$, the resulting spectral resolution in the Fourier spectrum is $\Delta f=f_{s}/L$ and the $k^{th}(k\in {\mathbb{Z}}^{+})$ spectral bin is associated with the frequency at $(k-1)f_{s}/L$. The interference from its counterpart in the imaginary part is unavoidable but if $f_{c}$ is located relatively far from the extremes of the positive axis marked at $f_{l}=0$ and $f_{h}=f_{s}/2$, the undesirable negative effect can be neglected. As such, the phase of the windowed signal at $f=f_{c}$ can be written as

(7) $$\varphi (k-k_{c})\approx \phi (k)-\theta .$$

However, it should be pointed out that the choice of k can also be a decimal besides the positive integer mentioned above.

**Figure 1:**
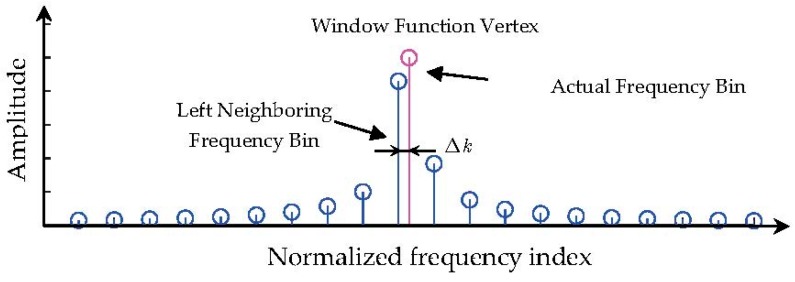
Illustration of some notations of energy leakage problem in FFT.

Additionally, we define the following useful concepts of frequency error (FE) with its normalized version when the digital signal is segmented with a specific type of window function. According to the fundamentals of signal processing, the actual single tone harmonic component is associated with the point with the maximal modulus in the spectral domain. We also name such a point a window vertex. Window vertexes may not be available in the fast Fourier Transform (FFT) spectrum because FFT only provides spectral bins evenly spaced at the spectral resolution Δf. As a result, frequency error, defined as the error between the actual frequency value and the frequency of its left neighboring spectral bin, occurs. This phenomenon can be observed in Figure 1. FE can be mathematically described as

(8) $$FE(f_{c}):=\left\lfloor \frac{f_{c}L}{f_{s}}\right\rfloor \cdot \frac{f_{s}}{L}-f_{c},$$

where ⌊ ⋅ ⌋ is a Gaussian function that rounds the input decimal number to the nearest integers towards minus infinity. Correspondingly, the normalized frequency error (NFE) of an actual frequency component in the FFT is defined as

(9) $$\Delta k:=\left\lfloor \frac{f_{c}L}{f_{s}}\right\rfloor -\frac{f_{c}L}{f_{s}}.$$

It is also known that Δk<0.

### 2.1. Principles of Ratio-Based Spectral Correction Methods

In this subsection, we mainly focus on a review of two classical ratio-based spectrum rectifying methods [29]. That is, one rectifying method using rectangular window and another using a Hanning window. Moreover, we show that both of these methods can run into severe problems in specific engineering circumstances.

#### 2.1.1. Rectangular-Window-Based Correction Method

The rectangular window of length L is defined by

(10) $$w_{r}(n)=1\;for\;n=0,1,2\ldots ,L-1.$$

Its spectral counterpart is expressed as

(11) $$W_{r}(w)=\frac{\sin(Lw/2)}{\sin(w/2)}e^{j(N-1)w/2},$$

where the modulus is $W_{r}(w)=\sin(Lw/2)/\sin(w/2)$. It is easy to know that

(12) $$W_{r}(w)=\frac{\sin(Lw/2)}{\sin(w/2)}e^{j(N-1)w/2},$$

because the spectral resolution of FFT is Δw=2π/L. The frequency associated with the kth spectral bin is w(k)=k⋅Δw=(2πk)/L for k=0,1,…,L/2−1. Therefore, the modulus function can be rewritten as

(13) $$W_{r}(w)=\frac{\sin(Lw/2)}{\sin(w/2)}e^{j(N-1)w/2}.$$

The approximation in Equation (13) is made on the assumption that the sampling length L is adequately large in value.

**Figure 2:**
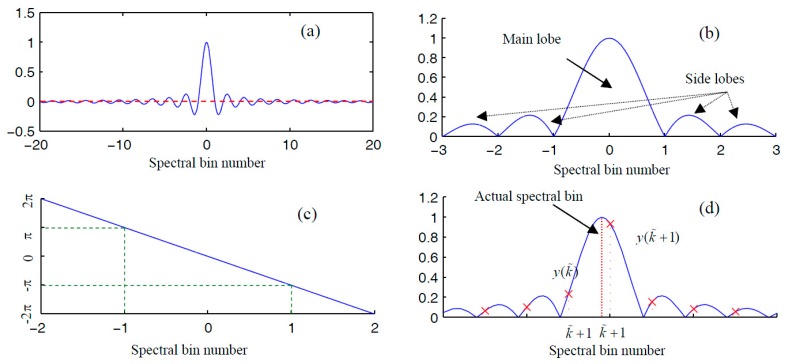
Fundamental principles of a rectangular-window-based spectral correction method. (**a**) The magnitude response; (**b**) zoomed-in plot of the magnitude response; (**c**) the phase response; and (**d**) the FFT grids compared with the magnitude response.

The normalized frequency width of a main lobe is 2. As such, for a sinusoidal component without exact positive period sampling, the accurate information of amplitude, frequency, and phase can be retrieved using the two neighboring spectral bins located within the main lobe around the actual frequency, which is shown in Figure 2.

Assume a simple harmonic component whose harmonic parameters are $\left\{A_{c},f_{c},\phi_{c}\right\}$ that is to be corrected in its FFT spectrum. Let $k_{c}$ be the precise decimal index associated with the actual harmonic component, Δk the normalized frequency error between the decimal index ($k_{c}=\left\lfloor (f_{c}\cdot L)/f_{s}\right\rfloor$) related to the actual frequency ($f_{c}$), and its left neighboring spectral bin marked by $\tilde{k}$. Therefore, the normalized frequency error Δk is defined as

(14) $$\Delta k=k_{c}-\tilde{k}.$$

For simplicity of explanation, a passenger function,

(15) $$T(\Delta k)=\frac{\sin(\pi (\Delta k))}{\pi (\Delta k)},$$

is introduced. It can be inferred that

(16) $$\begin{array}{cc} v & =\frac{y(\tilde{k})}{y(\tilde{k}+1)}=\frac{W(\Delta k)}{W(\Delta k+1)}\;\\ & =\frac{L\cdot \sin(\pi (-\Delta k))\cdot A_{c}}{\pi (-\Delta k)}\frac{\pi (-\Delta k+1)}{L\cdot \sin(\pi (-\Delta k+1))\cdot A_{c}}\;\\ & =\frac{T(\Delta k)}{T(\Delta k+1)} \end{array},$$

where $y(\tilde{k})$ and $y(\tilde{k}+1)$ are amplitudes of the two neighboring frequency bins around the actual one. Equivalently, the relationship in Equation (16) can be expressed as

(17) $$\tilde{k}\cdot T(\Delta k)+(\tilde{k}+1)T(\Delta k+1)=0.$$

In ratio-based spectral correction, Δk can be also expressed as

(18) $$\Delta k=-\frac{1}{1+v}=-\frac{y(\tilde{k}+1)}{y(\tilde{k})+y(\tilde{k}+1)}.$$

Hence, the index for the actual frequency can be obtained as $k_{c}=\tilde{k}-\Delta k$. In conclusion, the amplitude, frequency, and phase can be rectified as

(19) {amplitude=π(Δk)⋅T(k˜)sin(π(Δk))frequency=(k˜−Δk)fsLphase=arctanIm(k˜)Re(k˜)−π(Δk),

where Im(⋅) and Re[⋅] denotes the imaginary part and the real part of a complex-valued Fourier spectral bin.

#### 2.1.2. Cosine-Window-Based Correction Method

A cosine window is defined using the following mathematical prototype:

(20) $$w_{L}(n)=a-(1-a)\cos\left(\frac{2\pi n}{L}\right)\;for\;n=0,1,2\ldots ,L-1.$$

Correspondingly, its spectral counterpart is expressed as

(21) $$W_{L}(n)=\left\{a\cdot D(w)+\frac{1-a}{2}\left[D\left(w-\frac{2\pi }{L}\right)+D\left(w+\frac{2\pi }{L}\right)\right]\right\}e^{-jNw/2},$$

where D(w) represents the Dirichlet kernel defined as

(22) $$D(w)=\frac{\sin(Nw/2)}{\sin(w/2)}e^{\frac{jw}{2}}.$$

**Figure 3:**
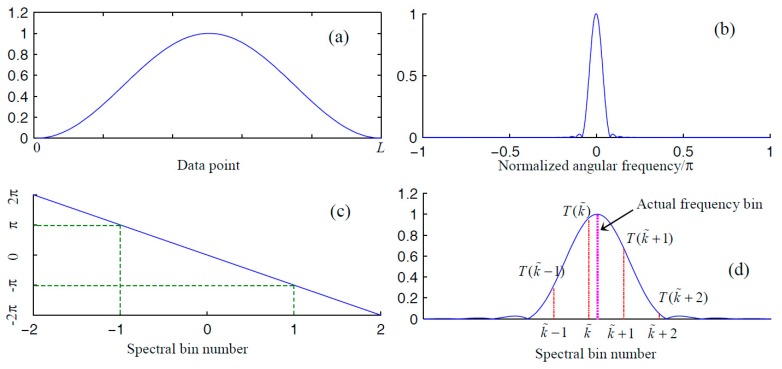
Fundamental principles of Hanning-window-based spectral correction method. (**a**) The magnitude response; (**b**) a zoomed-in plot of the magnitude response; (**c**) the phase response; and (**d**) the FFT grids compared with the magnitude response.

By setting parameter a in Equation (20) at 0.5, we can obtain a Hanning window. The modulus function of a Hanning window whose function vertex value is 1 (Figure 3a) can be deduced as

(23) $$M_{r}(k)=\frac{\sin(\pi k)}{\pi k}\cdot \frac{a+(1-2a)k^{2}}{1-k^{2}}.$$

The plot of modulus function in Equation (23) is shown in Figure 3b.

Without loss of generality, we illustrate high-precision spectral correction using the Hanning window. Let $\tilde{k}$ and $\tilde{k}+1$ be the indexes associated with the two neighboring frequency bins around the actual frequency. Using the notations introduced in Section 2.1, a passenger function with respect to normalized frequency error can be defined as

(24) $$T(\Delta k)=\frac{\sin(\pi (\Delta k))}{\pi (\Delta k)}\cdot \frac{1}{2(1-{\left(\Delta k\right)}^{2})}.$$

Substituting the variables $\tilde{k}$ and $\tilde{k}+1$ into Equation (24), we have the following relation:

(25) $$v=\frac{y(\tilde{k})}{y(\tilde{k}+1)}=\frac{T(\Delta k)}{T(\Delta k+1)}=\frac{\Delta k+2}{1-\Delta k}.$$

Equivalently, the above relation can be expressed as

(26) $$(\Delta k-1)T(\tilde{k})+(\Delta k+2)T(\tilde{k}+1)=0.$$

The normalized frequency error Δk can be deduced as below:

(27) $$\Delta k=\frac{T(\tilde{k}+1)-2T(\tilde{k})}{T(\tilde{k})+T(\tilde{k}+1)}.$$

Accordingly, the Hanning-window-based spectral correction for the harmonic information of amplitude, frequency, and phase can be summarized as

(28) {amplitude=πΔk⋅T(k˜)sin(πΔk)⋅2[1−(Δk)2]frequency=(k−Δk)fsLphase=arctanIm(k′)Re(k′)−πΔk.

### 2.2. Problems in Ratio-Based Spectrum Correction Methods

**Figure 4:**
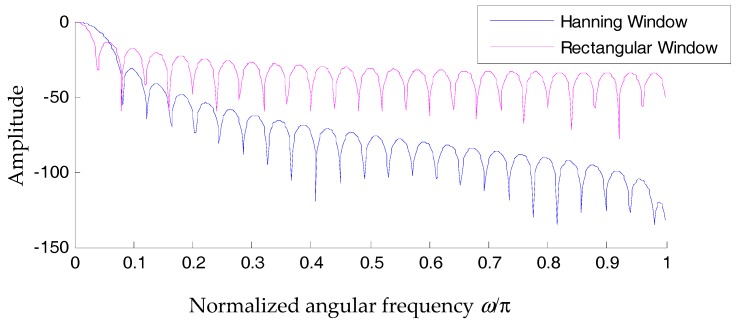
Comparison between the magnitude spectra of a rectangular window and the Hanning window.

Although ratio-based spectral correction can achieve high precision and adopts different kinds of window functions, a common characteristic is the utilization of complex-valued spectral bins located within the main lobe in the frequency domain. In a high signal-to-noise ratio (SNR) situation, for spectral correction problems of a single tone, the Hanning window is reported to be of higher precision compared with the rectangular window [29]. Magnitude responses of the two types of window functions are plotted in Figure 4.

From Figure 4, it is inferred that the main lobe bandwidth of the Hanning window is greater than that of the rectangular window. However, the magnitude of the side lodes of the latter decays much faster. Considering a simple harmonic signal whose frequency is exactly $f_{s}/4$, where $f_{s}$ is the sampling frequency, we can express the associated signal as

(29) $$x(t)=A\cos(2\pi f_{s}t+\phi ).$$

**Figure 5:**
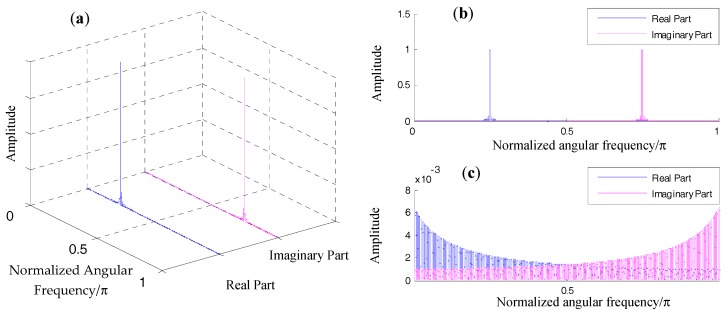
Demonstration of spectral aliasing of rectangular window: (**a**) magnitude response of the positive component and the negative component in a 3D coordinate; (**b**) magnitude response of the positive component and the negative component in a 2D coordinate; (**c**) zoomed-in plot of (**b**).

**Figure 6:**
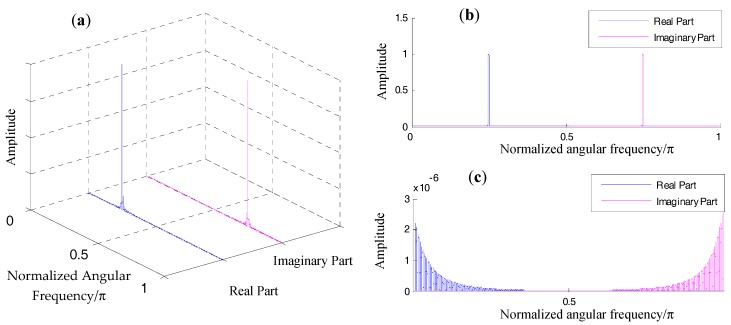
Demonstration of spectral aliasing of a Hanning window: (**a**) magnitude response of the positive component and the negative component in a 3D coordinate; (**b**) magnitude response of the positive component and the negative component in a 2D coordinate; (**c**) zoomed-in plot of (**b**).

For simplicity of comparison, we can set the amplitude A in the above equation at 1. In Figure 5a and Figure 6a, the spectra regarding the above signal when using different segmented windows are displayed.

A Hanning window offers better energy concentration in comparison with a rectangular window. However, no matter which type of window is employed, aliasing between the spectrum of the positive-frequency part and that of the negative-frequency part is inevitable. From Figure 5b, interferences between the two frequency parts are already visible on visual inspection. in Figure 6b, it can be observed that interferences between the two parts are almost negligible. To better illustrate their differences, zoomed-in plots of their aliasing effect are displayed in Figure 5c and Figure 6c. Because the rectangular window has a slow decaying rate, the amplitude at w=π/2 is approximately 0.002 of the amplitude at the window vertex (Figure 5c). In other words, these interferences are likely to affect the two spectral bins in the positive frequency axis that we used to correct the spectral bin information. As mentioned, a Hanning window decays much faster in the frequency domain. The amplitude at w=π/2 is approximately 10^–6^ of the amplitude at the window vertex.

In a noise-free situation, the above conditions explain why a Hanning window is superior to a rectangular window in rectifying a simple sinusoidal wave. However, problems regarding the correction precision would occur in both types of window functions when other interference components, for example the measurement noise, are presented in the recorded signals. The causes are listed below.

(1) As stated in Section 2.1, for spectral correction, both of the window functions employ two spectral bins, located in the main lobe. We refer to these spectral bins as main lobe spectral bins (MLSB). As any one of the MLSBs approaches the actual spectral bin, the other MLSB of rectangular will become small in amplitude. Mathematically, this phenomenon can be expressed as

(30) $$\underset{\Delta k\to 0}{\lim}\min\{y(\tilde{k}),y(\tilde{k}+1)\}=0$$

for the rectangular window and

(31) $$\underset{\Delta k\to 0}{\lim}\min\{y(\tilde{k}),y(\tilde{k}+1)\}=0.5$$

for the Hanning window. As a result, if Δk is small in value, one MLSB is likely to be corrupted even in the presence of a low level of noise.

(2) The higher the amplitude of an MLSB is, the greater noise resistibility is obtained. However, it is not possible to ensure that all the MLSBs are high in amplitude in the FFT grids when the normalized frequency error Δk is small.

## 3. The Proposed Active Frequency Shift Spectral Correction Method

### 3.1. Analysis of Condition Numbers of the Two Windowed Spectral Correction Methods

As explained in the above sections, the correctness of MLSBs plays an important role in guaranteeing precision results. It is expected that all MLSBs are large in amplitude such that each of them is equipped with robust noise resistibility. In this subsection, we attempt to investigate the energy distribution of the spectral bins located in the main lobe of the window function. The following concept of condition number for the MLSB set is defined. Let ${ℳ}_{rec}$ and ${ℳ}_{han}$ be the MLSB sets for the two types of window function, respectively. For the rectangular window, there are two elements in the set and the condition number is defined as

(32) $$CN_{rec}(\Delta k)=\frac{\min\left\{\left\|{ℳ}_{rec}\right\|\right\}}{\max\left\{\left\|{ℳ}_{rec}\right\|\right\}}.$$

For the Hanning window,

(33) $$CN_{han}(\Delta k)=\frac{\max\left\{\left\|MLSB\;\backslash \;\max\{\left\|{ℳ}_{han}\right\|\}\right\|\right\}}{\max\{\left\|{ℳ}_{han}\right\|\}}.$$

**Figure 7:**
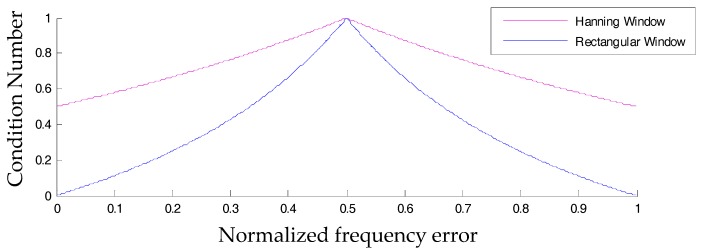
Condition numbers of rectangular window and Hanning window.

In the above definitions, the operator ||⋅|| computes the modulus of a complex-valued Fourier coefficient and the notation $MLSB(k)\backslash \max\{||{ℳ}_{han}||\}$ means eliminating the spectral bin with the maximal modulus form the original set. For a sinusoidal tone signal whose frequency is located near $f_{s}/4$ and does not belong to FFT grids, the plots of condition numbers regarding the two window functions with respect to Δk are displayed in Figure 7. As the condition number approaches unity in value, MLSBs have similar amplitude.

### 3.2. Algorithm of the Proposed Spectral Correction Technique

As indicated in Figure 7, the condition number of either type of window reaches the global maximum at Δk=0.5. This means that in such a situation, both of the two neighboring spectral bins around the actual frequency are high in amplitude. However, for the Hanning window, there are always two spectral bins relatively low in amplitude. Owing to these facts, we attempt to propose a novel active frequency shift spectral correction method based on a rectangular window.

Assuming that we have collected a digital signal x with *L* samples, the procedure of the proposed ASFFC algorithm is summarized as below. In the procedure, an index integer m is introduced and initialized as zero.

**Step 1.** Let $x^{\left(m\right)}(t)=x$, applying fast Fourier transform to the input signal.

(34) $$\hat{x}=FFT\{x\}.$$

**Step 2.** In the spectrum of the original signal, find some frequency ranges within which the amplitudes are large and are related to the characteristic frequency of the investigated mechanical system. A set of such spectral bins is denoted as

(35) $$V_{f}=\{f_{1},\ldots ,f_{M}\}.$$

The values of $f_{i}$ are local maxima in the Fourier spectrum. Choose the frequency bin of the greatest amplitude in $V_{f}^{\left(k\right)}$ and denote it as $\hat{f}$.

**Step 3.** Let the normalized integer index associated with the frequency $\hat{f}$ be denoted as $\hat{k}$; the value of the index variable $\tilde{k}$ in Section 2.1 is updated using the following formula:

(36) $$\tilde{k}:=\left\{ \begin{array}{ccc} \hat{k} & for & T(\hat{k}+1)\geq T(\hat{k}-1)\\ \hat{k}-1 & for & T(\hat{k}+1)<T(\hat{k}-1) \end{array}\right..$$

**Step 4.** Compute the harmonic information of two MLSBs, which are denoted as $\left\{A(\tilde{k}),f(\tilde{k}),\phi (\tilde{k})\right\}$ and $\left\{A(\tilde{k}+1),f(\tilde{k}+1),\phi (\tilde{k}+1)\right\}$.

**Step 5.** Correct the harmonic information using the two MLSBs in Step 4 via a ratio-based spectral correction method using a rectangular window. Let the corrected information of frequency be $A^{\prime }(\tilde{k})$.

**Step 6.** Compute some interpolated spectral bins using the original definition of DFT. Let the normalized frequency increment δ=0.1; the set of the zoomed-in spectral grid $S_{\hat{k}}$ is defined as

(37) $$\begin{array}{cc} S_{\hat{k}}=\{\tilde{k}, & \underset{spectral\;\mathrm{bins}\;\mathrm{between}\;\tilde{k}\;and\;\tilde{k}+1}{\underset{︸}{\tilde{k}+\delta ,\tilde{k}+2\delta ,\ldots ,\tilde{k}+9\delta }},\tilde{k}+1,\\ & \;\underset{spectral\;\mathrm{bins}\;\mathrm{between}\;\tilde{k}+1\;and\;\tilde{k}+2}{\underset{︸}{\tilde{k}+1+\delta ,\tilde{k}+1+2\delta ,\ldots ,\tilde{k}+1+5\delta }}\} \end{array}$$

for $A(\tilde{k}+1)\geq A(\tilde{k})$, and

(38) $$\begin{array}{cc} S_{\tilde{k}}= & \{\underset{spectral\;\mathrm{bins}\;\mathrm{between}\;\tilde{k}-1\;and\;\tilde{k}}{\underset{︸}{\tilde{k}-5\delta ,\tilde{k}-4\delta ,\ldots ,\tilde{k}-\delta }},\\ & \;\tilde{k},\;\underset{spectral\;\mathrm{bins}\;\mathrm{between}\;\tilde{k}\;and\;\tilde{k}+1}{\underset{︸}{\tilde{k}+\delta ,\tilde{k}+2\delta ,\ldots ,\tilde{k}+9\delta }},\tilde{k}+1\} \end{array}$$

for $A(\tilde{k}+1)<A(\tilde{k})$.

**Step 7.** Find two spectral bins in the set $S_{\tilde{k}}$ such that

(39) $$\ell =\arg\underset{\ell \in S_{\tilde{k}}}{\min}|T(k+\ell \cdot \delta )-T(k+(\ell +10)\delta )|.$$

**Step 8.** Apply ratio-based spectral correction algorithm as we introduced in the rectangular window case using the two selected spectral bins (Equations (18) and (19)). As a result, the corrected harmonic information $\left\{f^{\prime }(\tilde{k}),\phi^{\prime }(\tilde{k})\right\}$ is obtained.

**Step 9.** Synthesize a simple sinusoidal signal using the obtained corrected harmonic information

(40) $$s_{\hat{k}}(t)=A^{\prime }(\tilde{k})\cos(2\pi f^{\prime }(\tilde{k})t+\phi^{\prime }(\tilde{k}))$$

and subtract $s_{\hat{k}}(t)$ from $x^{\left(m\right)}(t)$.

**Step 10.** Let $x^{\left(m+1\right)}(t)=x^{\left(m\right)}(t)-s_{\tilde{k}}(t)$ and m=m+1 m. Eliminate $\tilde{f}$ from $V_{f}$ and go back to Step 2. The iterative procedure ends when all the sinusoidal components are corrected.

**Figure 8:**
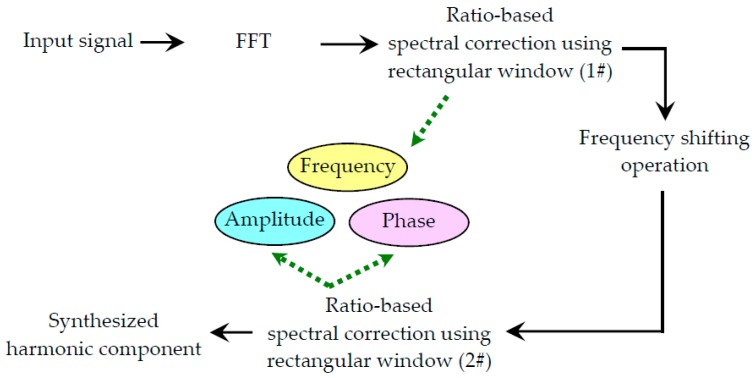
The interpolated spectral bins around an actual harmonic component.

The above procedure can be divided into two parts. Step 6 refers to the active frequency shifting operation, which is capable of enhancing the noise resistibility of spectral bins in the subsequent spectral correction steps. Step 7 is the classical spectral correction using a rectangular window. It should be mentioned that the information of frequency is corrected prior to the frequency shifting operation and the information of amplitude and phase is estimated after the frequency shifting operation, as illustrated in Figure 8. Reasons for the proposed algorithm are explained in Section 4.

### 3.3. Condition Number Analysis after Frequency Shifting Operation

**Figure 9:**
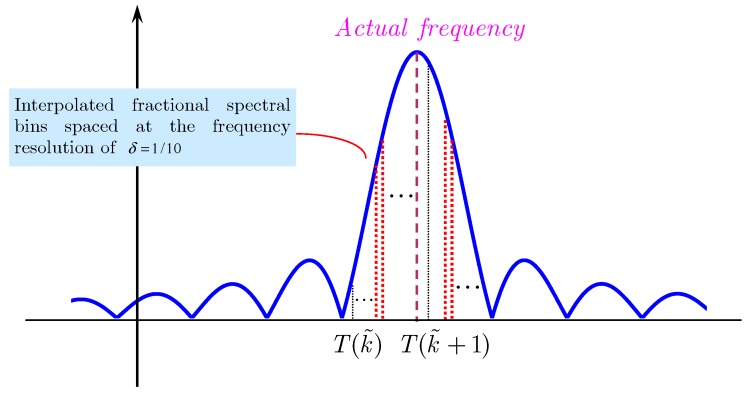
The interpolated spectral bins around an actual harmonic component.

The interpolated spectral bins around an actual harmonic component are demonstrated in the schematic diagram of Figure 9. The spectral interpolation step can also be combined with ratio-based spectral correction using a Hanning window.

**Figure 10:**
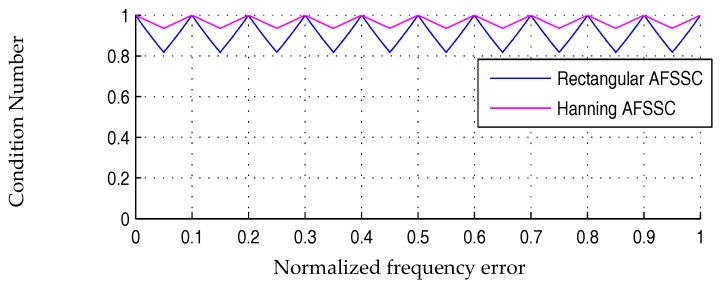
Condition numbers of rectangular-window-based and Hanning-window-based spectral correction methods enhanced by spectral interpolations.

Curves of condition numbers belonging to a rectangular window as well as a Hanning window, after the spectral interpolation step, are displayed in Figure 10. Serving as a post-processing process after FFT, the active frequency shifting operation can also be combined with ratio-based spectral correction using a Hanning window. Therefore, four spectral correction methods are available. The abbreviations of the proposed method and those of the comparison methods are listed in Table 1.

## 4. Numerical Analysis of the Proposed AFSSC Method

In this section, we attempt to verify the enhancement of the proposed AFSSC method in retrieving harmonic information corrupted by strong noises.

### 4.1. Performance Comparison in the Presence of Noises

Assuming that a record of measured simple sinusoidal signal, corrupted by white Gaussian noise wgn(t), is synthesized as below:

(41) $$s(t)=\cos(2\pi f_{c}t+\phi )+wgn(t),$$

where ϕ is a variable representing the phase of the signal. The sampling frequency $f_{s}$ of the simulated signal is 1000 Hz. The sampling length is 1000 for the signal. Correspondingly, the spectral resolution of s(t) via FFT is calculated at 1 Hz. The value of $f_{c}$ is located in the positive frequency part not belonging to spectral grids generated by FFT.

#### 4.1.1. Tests when the Frequency and the Amplitude of the Signal Are Both Fixed.

**Figure 11:**
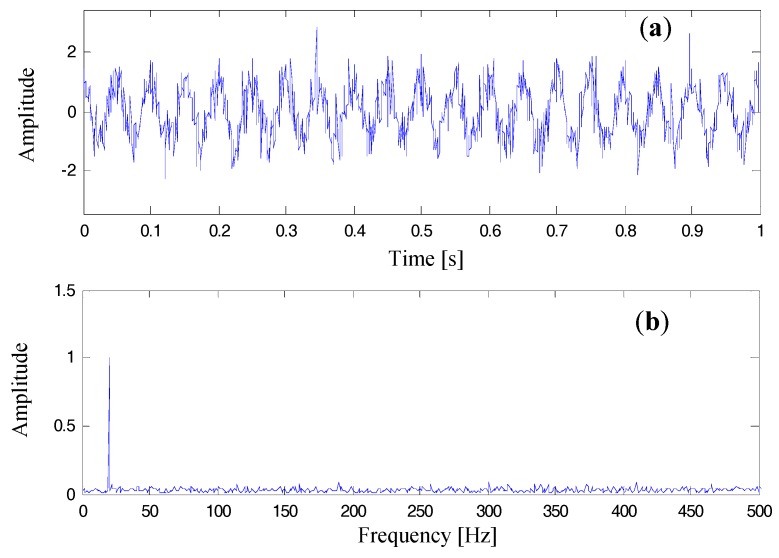
Plots of the simulated sinusoidal wave corrupted by white Gaussian noise (SNR = 3dB): (**a**) time domain wave; (**b**) FFT spectrum.

Let the frequency of the harmonic component in Equation (41) be set to 20.01 Hz. Owing to the fact that the spectral resolution of s(k) is 1 Hz, the frequency of the spectral bin with the greatest modulus in the corresponding FFT spectrum is calculated at 20 Hz. Hence, the normalized frequency error of using FFT to estimate $f_{c}$ is Δk=0.01. The amplitude of the single-tone signal is set to 1. In the following independent spectral correction tests, the parameter of ϕ is discretized in the real-valued interval of [0,π] with a phase increment π/1000. That is to say, 1000 independent tests were implemented for each parameter combination. In the following tests, all the values of signal ($\cos(2\pi f_{c}\cdot t+\phi )$) to noise (wgn(t)) ratio are set to 3 dB. A typical example of s(t) with its Fourier spectrum $\hat{x}(f)$ is shown in Figure 11.

**Figure 12:**
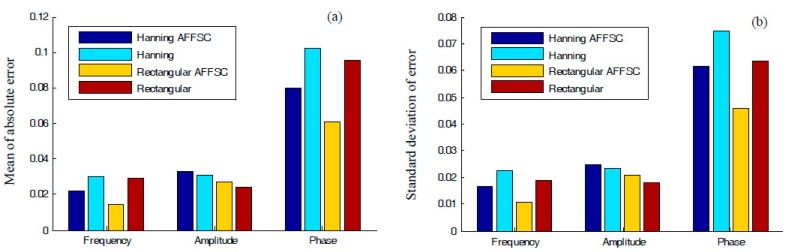
(**a**) Mean values of the estimated errors regarding the harmonic information (frequency, amplitude, and phase) using different spectral correction methods, and (**b**) standard deviations of the estimated errors regarding the harmonic information (frequency, amplitude, and phase) using different spectral correction methods.

In Table 2, the correction results of the four comparison methods (HanRB, HanAFSSC, RecRB, and RecAFSSC) are listed. Mean values and standard deviations of the absolute correction errors are computed, displayed in the bar charts in Figure 12. Among the results, RecAFSSC exhibits the highest precision in correcting the harmonic information of frequency and phase. As for the amplitude, RecRB achieves the highest precision and RecAFSSC is only inferior to RecRB.

As the analyzing frequency of the simulated signal is 500 Hz, which is half of the sampling frequency according to the Shannon’s information theory of sampling, the parameter $f_{c}$ can also be properly set at 250.01 Hz and 480.01 Hz. Tests with a similar procedure to that mentioned above are conducted. Corresponding results, which are listed in Table 3 and Table 4, maintain the same conclusions as we have obtained in the case when $f_{c}=20.01\mathrm{Hz}$.

#### 4.1.2. Tests when the Amplitude of the Harmonic Is Fixed

**Figure 13:**
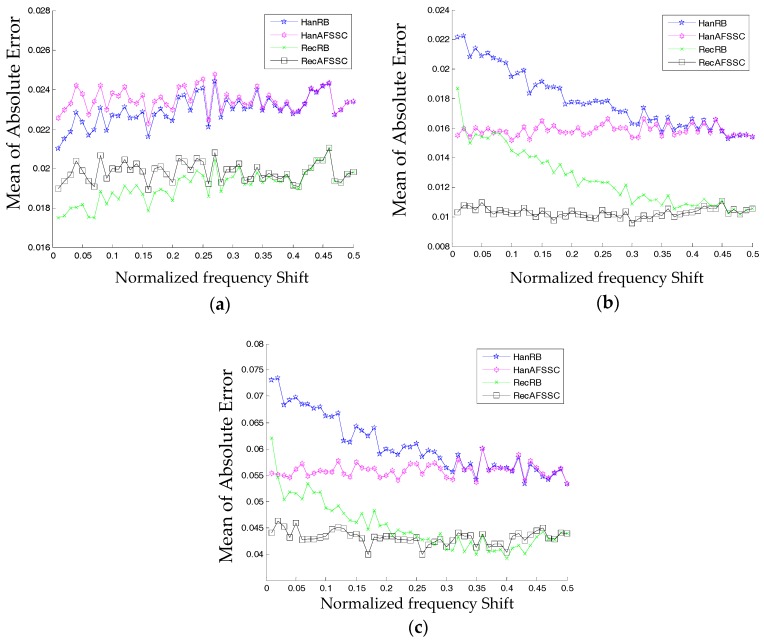
Comparisons on the comparison of four comparison methods with respect to information of (**a**) amplitude, (**b**) frequency, and (**c**) phase.

In the following tests, instead of selecting a fixed frequency shift, we explore the correcting performance of the proposed AFSSC method when Δk changes continuously in the normalized frequency range (0, 0.5). As shown in Table 2, Table 3 and Table 4, since the change of $f_{c}$ does not affect the conclusion of the results, we can set $f_{c}$ at (249+Δk) Hz, in which Δk∈(0,0.5). The curves in Figure 13 give detailed comparisons of the four methods with respect to amplitude, frequency, and phase.

The Y axis in Figure 13 is the mean of the absolute error of some specific harmonic information. As indicated in Figure 13a,b, it can be inferred that (*i*) RecRB offers the optimal performance in correcting the information of amplitude, especially when Δk is small, and (*ii*) RecAFSSC is superior in correcting the frequency no matter what the value of Δk is. Moreover, RecAFSSC is also of highest precision in correcting the phase information when Δk is small (Δk∈(0,0.3), as shown in Figure 13b. When Δk∈[0.3,0.5], the performance of RecRB outperforms the proposed RecAFSSC in correcting the phase information. However, the improvement of the former over the latter is quite small. We can also say that these two methods are of equal accuracy for Δk∈[0.3,0.5]. In conclusion, simulations in this subsection have clarified the enhanced performance of the proposed AFSSC technique. The proposed technique actually combines the advantages of RecRB and RecAFSSC. Considering the information in Figure 7, Figure 10 and Figure 13, we can also conclude that (*i*) for higher values of CN, attained via the active frequency shifting, operation is beneficial for enhancing the correction accuracies of frequency and phase, and (*ii*) higher values of CN can reduce the correcting accuracy of amplitude.

### 4.2. Discussion of the Proposed AFSSC with Classical High-Precision Harmonic Information Correcting Algorithms

**Figure 14:**
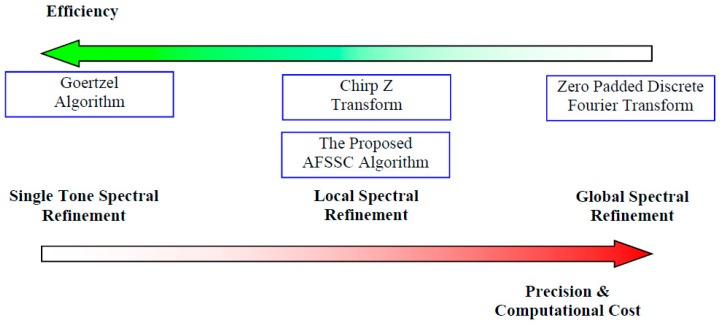
Pros and cons of some classical spectral refinement techniques with the proposed AFSSC algorithm.

As mentioned in Section 1, some classical spectral refinement techniques such as zero-padded DFT, chirp Z transform, and the Goertzel algorithm are also helpful to correct the spectrum in the presence of strong masking noises. To some extent, the essence of the above methods is identical. That is, they attempt to approximate the window vertex, which represents the almost precise spectral bin of the original signal, in different numerical ways. The mathematical principles behind them can be uniformly summarized as discrete Fourier-transform-based spectral interpolation. In the proposed method, Step 4 in Section 3.2 can be regarded as an equivalent realization of CZT around the main lobe of the window function at a refined normalized spectral resolution, Δk=0.1. In this equivalent spectral refinement process, 15 additional spectral bins are interpolated to enhance the accuracy of the related harmonic information. Although the 15 spectral bins are derived using DFT, this process can also be speeded up via the Goertzel algorithm, which is especially efficient in generating spectral bins with known frequency values. Zero-padded DFT, implemented via FFT algorithm, can make a refined Fourier spectrum with extreme precision. It is especially suitable for correction harmonic information of signals containing many single-tone components. However, for zero-padded DFT, high precision is attained at the expense of great computational cost. In Figure 14, we vividly illustrate the differences among the various kinds of spectral refinement methods as well as the proposed AFSSC algorithm. AFSSC is proposed in combination with local spectral refinement with a ratio-based spectral correction method. The former can acquire spectral bins with proper noise resistibility and the latter can estimate the actual harmonic information with high efficiency.

## 5. Application of AFSSC in a Case Study of Crack Growth Detection in Bladed Machinery of BFG Power Plant

**Figure 15:**
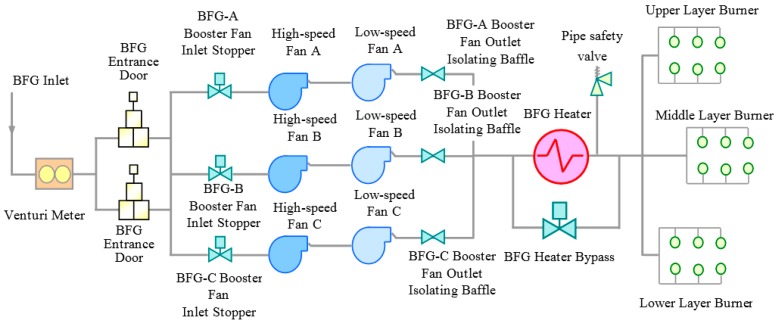
Schematic diagram of the BFG power plant.

In this section, a case study regarding the structural health monitoring of a BFG power plant is introduced. The project of this power plant was launched by a major manufacturer that supplies high-end steel products in China. As this manufacturer is located in a major metropolis of eastern China, there is high demand regarding environmental protection issues. This power plant was established to promote exhaustive burning of environmentally harmful BFGs such that residual energies can also be recycled for steel product manufacturing. A schematic diagram of the BFG power plant is given in Figure 15.

The installed capacity of this power plant is 350 MW. In addition to BFG, alternative input fuels of the power plant can also be coke oven gas (COG), corex gas (or other industrial gas), natural gas, or light oil. The power plant is world-renowned for its combustion boiler. The maximal flow rate of the boiler is 1131 km^3^/h, the highest burning flow rate in the world in 2011. As indicated in Figure 15, the fueling system has three dual-speed booster fan units. As the key component of the booster fans, the centrifugal compressor can operate at a lower speed of 744 r/min (12.4 Hz) and a higher speed of 993 r/min (16.55 Hz). The tower house boiler belongs to the type of micro-positive pressure combustion, which is equipped with 18 composite gas burners. These burners are deployed at three distinct layers. As the power plant is an important asset, routine inspections regarding its health state were conducted.

### 5.1. Failure Case Description

**Figure 16:**
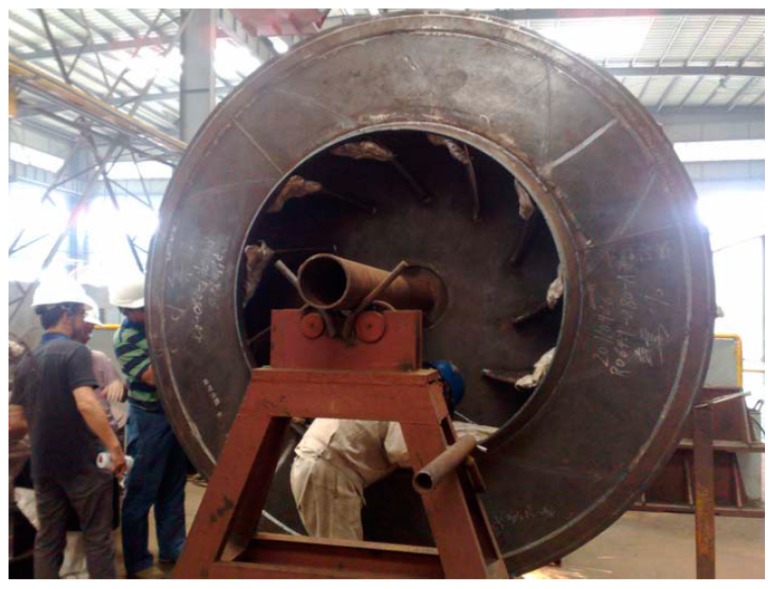
Photograph of the centrifugal compressor with a fully developed blade crack.

An accident occurred on the booster fan of this BFG power plant. This accidental event was caused by a broken blade on the centrifugal compressor. There are 11 impeller blades arranged in the radial direction of the centrifugal compressor. The fault source was found to be a fully developed crack on the faulty impeller blade (Figure 16), which reduced the mechanical strength between the faulty blade and the impeller base.

**Figure 17:**
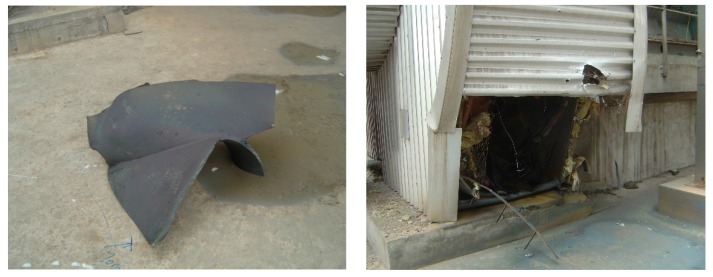
Photograph of destroyed housing of the BFG booster.

The faulty blade was thrown away due to strong centrifugal force at a time when the booster fan was operating at the lower speed (744 r/min). The faulty blade knocked against the volute and caused severe structural damage. A piece of volute debris even broke the housing of the booster fan, as shown in Figure 17. Fortunately, there were no casualties caused by this fault. However, to prevent the future occurrence of such accidents, condition monitoring of this machinery should be strengthened.

### 5.2. Monitoring Data Analysis Using the Proposed AFSSC Method

**Figure 18:**
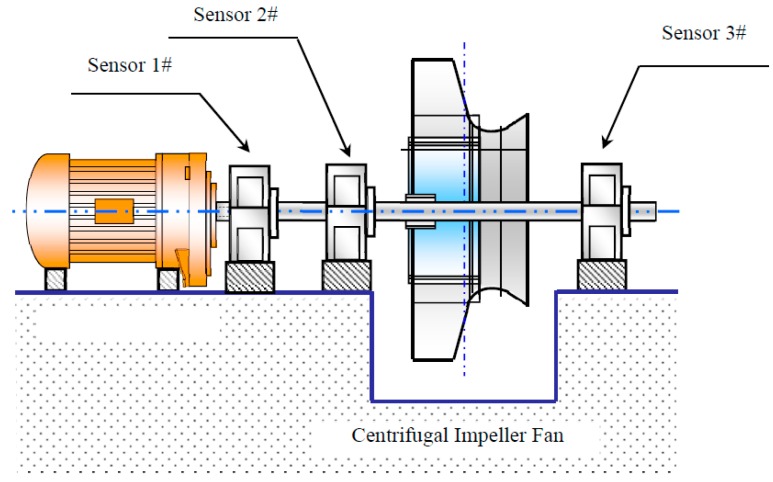
Mechanical transmission chain of the booster fan (from the driving AC motor to the centrifugal compressor).

**Figure 19:**
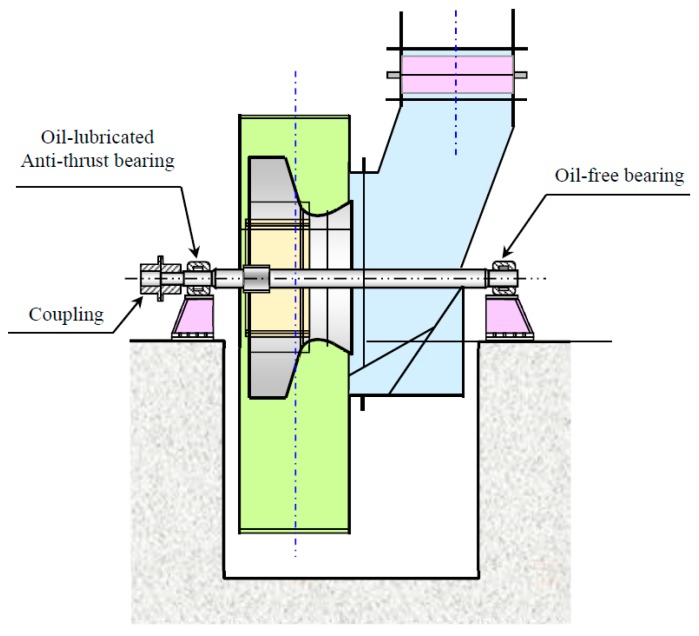
Detailed schematic diagram of the booster fan and its key component, the centrifugal compressor.

The mechanical transmission chain of the booster fan is illustrated in Figure 18. A more detailed description of the centrifugal compressor is given in Figure 19.

As shown in Figure 18 and Figure 19, as the key component of the booster fan, the centrifugal compressor is driven by an AC motor via a rigid coupling. There are three bearing housings from the end of the motor output to the right end of the centrifugal compressor. Vibration sensors were deployed on the bearing housings due to the convenience of accessibility. In each sensor point, vibrations in three directions (axial, horizontal, and vertical) were measured. Signals collected from the axial directions are acceleration signals and signals from the horizontal direction are velocity signals. The acronym GE in Table 5 refers to the envelope amplitude of acceleration signals. It was acquired via hardware integrations in the data acquisition appliance. Because location 2 and location 3 are closer to the centrifugal compressor, we focus on analyzing the signals collected from them.

To investigate the historical measurements of this faulty booster fan, the collected vibration signals stored in the factory database were checked. There are 10 sets of historical data that can be traced. The data, along with the scheme of each measurement, are listed in Table 5. The earliest test was conducted about 11 months before the accidental event. For the historical measurements, the booster fan operated at the lower speed (744 r/min, indicated by letter ‘L’) eight times and the higher speed (993 r/min, indicated by letter H) two times. Because their working speeds are not exactly the same, this adds to the difficulty of the signal analysis. Among these measurements, the sample frequency and the sampling length in each channel are uniformly set at $f_{s}=2560$ and L=4096.

### 5.3. Statistical Indicator Analysis of the Vibration Data

In order to make an overall analysis of the vibration measurement, a statistical analysis was performed. Two indicators, the root mean square (RMS) value and the kurtosis in the time domain, were used. RMS can detect the energy amplitude of the time series, while kurtosis can measure the peakiness of the potential impact components. The two indicators for discrete series x of length *L* are defined as

(42) $$RMS\{x\}={\left(\frac{\sum_{k=0}^{L-1}x^{2}(k)}{L}\right)}^{1/2}$$

(43) $$\mathrm{Kurtosis}\{x\}=\frac{\mu_{4}}{\sigma^{4}}=\frac{E[{\left(x-\mu \right)}^{4}]}{{\left(E[{\left(x-\mu \right)}^{2}]\right)}^{2}},$$

where $\mu_{4}$ is the fourth central moment and σ is the standard deviation.

**Figure 20:**
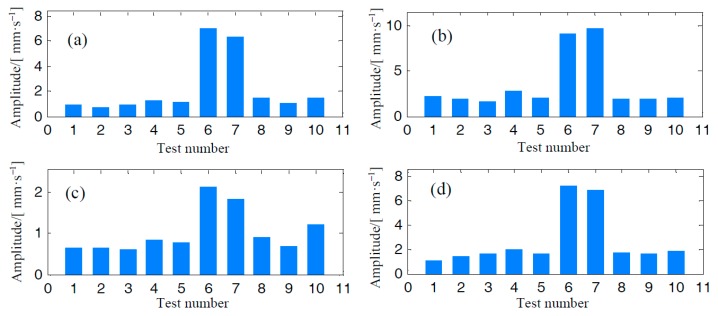
RMS trends of the centrifugal compressor: (**a**) Channel ‘2A’, (**b**) Channel ‘2H’, (**c**) Channel ‘3A’, and (**d**) Channel ‘3H’.

In Figure 20, we plot the trends of RMS values according to the data from channels denoted as ‘2A’, ‘2H’, ‘3A’, and ‘3H’. The concept of vibration severity (VS), indicated by the RMS value, is introduced to describe the mechanical vibration level of the centrifugal compressor. It is observed that VS at high speed states are generally much bigger than those at low speed states. However, there is no explicitly positive trend growth regarding the RMS values computed via signals from the eight measurements at low speed states. As indicated by the sub-figures, many VS indicated by ‘8~10’, measured at dates closed to the date of the mentioned accidental event, are even smaller than those measured at very early dates.

**Figure 21:**
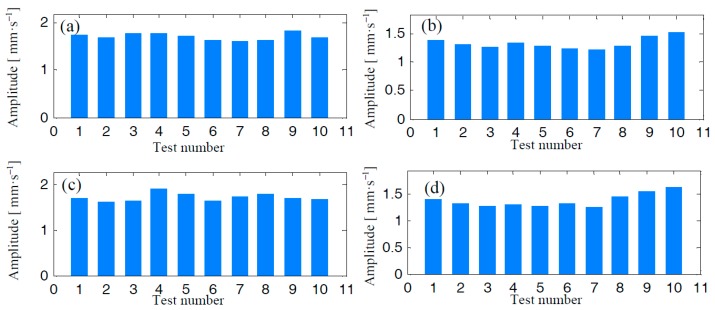
Kurtosis trends of the centrifugal compressor: (**a**) Channel ‘2A’, (**b**) Channel ‘2H’, (**c**) Channel ‘3A’, and (**d**) Channel ‘3H’.

In Figure 21, we also display the trends of kurtosis values according to the data at channels denoted as ‘2A’, ‘2H’, ‘3A’, and ‘3H’. No matter what the operation speed is, there is no explicit growth trend regarding kurtosis values. The kurtosis values of measurements at high speed are even smaller than those at low speeds.

According to the results revealed by Figure 20 and Figure 21, classical statistical analysis failed to detect cracks during the duration of crack growth. The only finding is that the operation speed has a significant influence on the vibration severity of the centrifugal impeller.

### 5.4. Crack Feature Analysis via the Proposed AFSSC Method

According to the materials presented above, the rotation frequency of the centrifugal impeller at low working speed can be computed as

$$f_{wl}=\frac{745}{60}\approx 12.4167(\mathrm{Hz})$$

and the rotation frequency at high speed can be computed as

$$f_{wh}=\frac{993}{60}\approx 16.5500(\mathrm{Hz}).$$

The frequency resolution of the vibration measurement is

$$\Delta f=\frac{2560}{4096}=0.625(\mathrm{Hz}).$$

This means that we can obtain frequency bins, related to rotation frequencies form the Fourier spectra, at 12.5 Hz and 16.25 Hz. From the fast Fourier spectra, the estimated working frequencies are ${\tilde{f}}_{wl}=12.5\mathrm{Hz}$ and ${\tilde{f}}_{wl}=16.25\mathrm{Hz}$. The related normalized frequency error are $\Delta_{wl}=0.0133$ and $\Delta_{wh}=0.48$. That is to say, there will be significant numerical errors in the estimated harmonic information of amplitude, frequency, and amplitude when the original FFT spectra are investigated. Therefore, we employ the proposed active frequency shift spectral correction method. Let X denote the fundamental rotation frequency and *i*-X denote the corresponding *i*th-order harmonic component of the fundamental working frequency.

**Figure 22:**
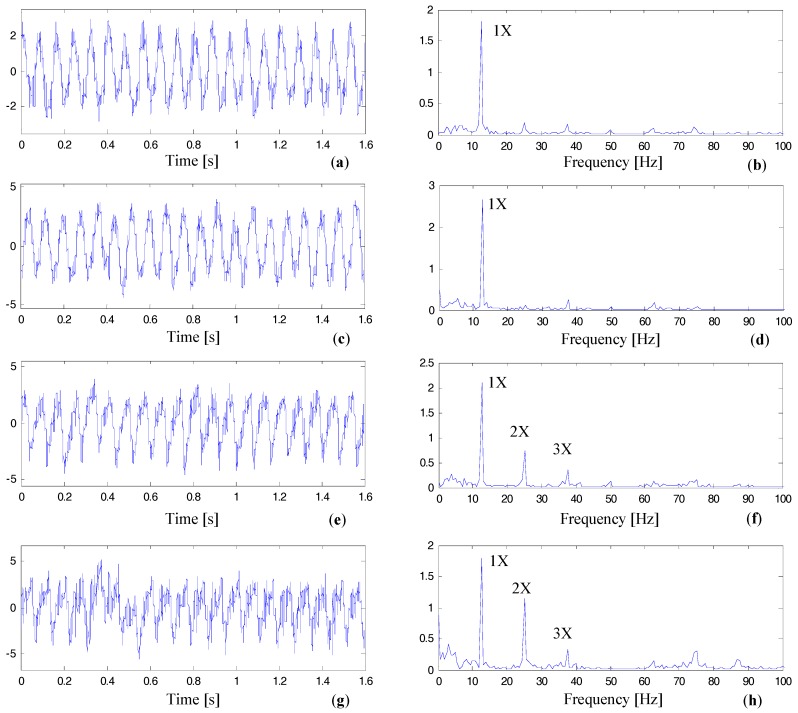
Time domain waves and FFT spectra of the vibration signal belonging to sensor point 3H: (**a**) the time domain wave of Test No. 2, (**b**) Fourier spectra of Test No. 2, (**c**) the time domain wave of Test No. 4, (**d**) Fourier spectra of Test No. 8, (**e**) the time domain wave of Test No. 8, (**f**) Fourier spectra of Test No. 8, (**g**) the time domain wave of Test No. 10, (**h**) Fourier spectra of Test No. 10.

In Figure 22, we demonstrate the time domain wave and the FFT spectral of vibration signals collected from the horizontal channel at sensor point 3. We mainly focus on spectra analysis of the lower frequency range, which is [0,100]Hz. As can be seen, information from the four tests are sufficient to reveal growth trends in two distinct domains (Test No. 2, Test No. 4, Test No. 8, and Test No. 10 marked in Table 5. In the time domain, there are more nonstationary components as we approach the date of the accidental event. Importantly, the FFT spectra give us more valuable information. Although working loads of each test are different, the amplitudes of 2× and 3× are low in early tests (Figure 22b,d). The energy of the fundamental frequency is dominant in the frequency range of [0,100]Hz. As the time evolves, there is significant growth for the amplitudes of 2× and 3× (Figure 22f,h).

As such, in the following analysis, attention is focused on the information of 1×, 2×, and 3×. Using the proposed AFSSC, corrected information regarding amplitude, frequency, and phase is listed in Table 6.

To give a precise description of the information, we utilize the concept of a simple harmonic phasor to incorporate the corrected spectrum information $\left(j,A_{i,j},∡\phi_{i,j}\right)$. That is, let the *j*th order harmonic component in the *i*th vibration test be denoted by

$$E_{i,j}(t)=A_{i,j}\cdot \cos(2\pi f_{i,j}t+∡\phi_{i,j})\;,\;t\in [0,1.6]$$

where $A_{i,j}$, $f_{i,j}$, $∡\phi_{i,j}$ are the corrected amplitude, the corrected frequency, and the corrected phase of $E_{i,j}(t)$, respectively. Therefore, in the *i*th test, the velocity signal V(t) containing harmonic components of 1×, 2×, 3× can be written as below:

$$\begin{array}{cc} V_{i}(t) & =E_{i,1}(t)+E_{i,2}(t)+E_{i,3}(t)\\ & =A_{i,1}\cdot \cos\left[2\pi f_{i}t+∡\phi_{i,1}\right]\;+A_{i,2}\cdot \cos\left[2\pi (2f_{i})t+∡\phi_{i,2}\right]+A_{i,3}\cdot \cos(2\pi (3f_{i})t+∡\phi_{i,3})\;,\;t\in [0,1.6] \end{array}$$

Because the dimension of the original vibration signal is velocity (mm/s), an operation of time domain derivative is required to transform $V_{i}(t)$ into its acceleration version $A_{i}(t)$ such that the

$$\begin{array}{cc} A_{i}(t)=\frac{dV_{i}(t)}{dt}= & -A_{i,1}\cdot f_{i}\cdot \sin\left[2\pi f_{i}t+∡P_{i,1}\right]\\ & \;-A_{i,2}\cdot 2f_{i}\cdot \sin\left[2\pi (2f_{i})t+∡P_{i,2}\right]\\ & \;-A_{i,3}\cdot 3f_{i}\cdot \sin\left[2\pi (3f_{i})t+∡P_{i,3}\right]\;t\in [0,1.6] \end{array}$$

For the acceleration signal $A_{i}(t)$, we propose a normalized health indicator ${Weight}_{i,j}$ with respect to each phasor $E_{i,j}(t)$, which can be deduced using corrected harmonic information shown as below:

$${Weight}_{i,j}=\frac{j\cdot A_{i,j}}{A_{i,1}+2A_{i,2}+3A_{i,3}}\times 100\%\;,\;j=1,2,3.$$

**Figure 23:**
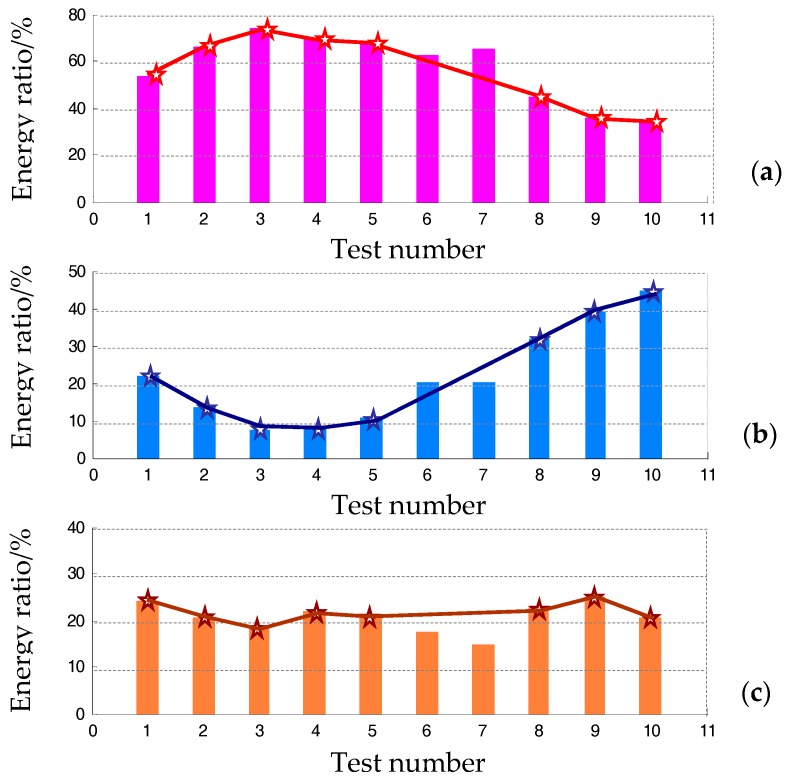
Energy weight of the three harmonic tones of the fundamental working frequency: (**a**) the first order, (**b**) the second order, and (**c**) the third order.

The associated energy weights are shown in Figure 23. Although the information of vibration tests at different rotation speed may be incomparable, we still plot them in an identical coordinate for each harmonic component of *j*th-X. As for the fundamental frequency, the normalized energy weight became larger from Test No. 1 to Test No. 3, and this indicator decreased in later tests. Remarkably, values of the energy weight of the second order decreased in early tests and increased significantly in later tests. There is no obvious growth trend regarding the third-order harmonic component.

From the above results, it is concluded that the growth in the amplitude of higher-order harmonic component successfully indicates the evolution of a dynamic imbalance in the centrifugal impeller. Therefore, the normalized indicator, proposed based on the AFSSC technique, can serve as an effective measure of the crack growth in the blade.

### 5.5. Comparisons

To validate the enhancement of the proposed AFSSC in harmonic information recovery, we also employ FFT and ratio-based spectral correction method to process the signals.

If we interpret the harmonic information by merely using FFT, there is no correction of amplitude or phase and the spectral bins with maximal modulus around the harmonic tones are investigated. Following the procedure introduced in Section 5.4, the normalized health indicator in the form of energy ratio with respect to each harmonic tone (1×, 2×, and 3×) is shown in Figure 23.

**Figure 24:**
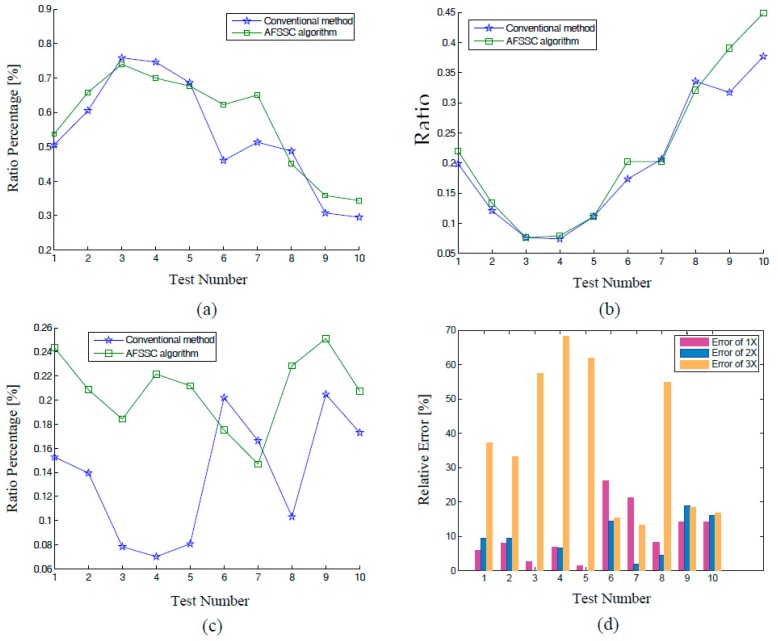
Comparison between the results of AFSSC and those of FFT: the first order, (**b**) the second order, and (**c**) the third order; and (**d**) curves of relative errors of each harmonic tone in each test.

To make a comparison, results in Figure 23 are also reproduced simultaneously in Figure 24. Let the relative error of a *j*th order single-tone component in the *i*th test be defined as

(44) $${RE}_{i,j}=\frac{\left|{Weight}_{i,j}-{\bar{Weight}}_{i,j}\right|}{{Weight}_{i,j}}\times 100\%\;,$$

where *j* = 1, 2, 3 and ${\bar{Weight}}_{i,j}$ indicates the energy ratio estimated via FFT.

In the early tests, the amplitude of the components 2× and 3× are low in energy, therefore the error between ${Weight}_{i,j}$ and ${\bar{Weight}}_{i,j}$ is relatively small, as can be observed in Figure 24a,b. As the crack on the impeller blade developed, the differences became much greater in later tests. It should be noticed in Figure 24b that the curve of the energy weight of 2× no longer exhibits a monotonic increasing trend, which suggests the occurrence of a large estimation error. In Figure 24c, the difference between ${Weight}_{i,j}$ and ${\bar{Weight}}_{i,j}$ is so large that the results of ${\bar{Weight}}_{i,j}$ regarding the harmonic component 3× are not accurate at all. Moreover, we plot curves of relative error with respect to each harmonic tone of each test (Figure 24d).

## 6. Discussions on Potential Applications of AFSSC in Materials Engineering

In this paper, we propose the AFSSC algorithm to address spectral correction problems in the field of structural health monitoring. AFSSC achieves a good trade-off between efficiency and accuracy. It is suitable for correcting harmonic information from signals of relative short sampling length. To the authors’ knowledge, there have been many studies employing FFT and its related spectral analyzing techniques. Such techniques are often adopted to investigate the physical behaviors of materials. Li and Shen applied FFT as an alternative approach to the finite element method to compute the effective properties of composite materials with a periodic microstructure [37]. Nicholas and Marko presented a computational procedure based on fast Fourier transforms to delineate elastic property closures for hexagonal close-packed metals [38]. In their research, a database of non-zero Fourier transforms is built for each component of the elastic stiffness tensor. Lu and Xu utilized Fourier transform infrared spectra to characterize the properties of hybrid particles [39]. Since AFSSC is presented as an enhancement to DFT, FFT as well as zoomed-in spectral interpolation, it will also be applicable in similar research to retrieve delicate spectral information from the measured dynamic processes. We believe that it will find more applications in the future.

## 7. Conclusions

In this paper, we investigate crack detection problem of blade machinery using vibration-based condition monitoring techniques. The major findings are summarized as follows:

(1) A novel spectral correction method is proposed. Rather than using spectral bins of the FFT spectrum for correcting all harmonic information (amplitude, frequency, and phase), the proposed AFSSC method utilizes zoomed-in spectral interpolations around the actual harmonic components. The amplitudes of the two spectral bins, employed for ratio-based spectral correction, are high in value such that robust noise resistibility is ensured. By utilizing the active frequency shifting operation, the values of CN are increased, which is beneficial for enhancing the correction accuracies of frequency and phase.
(2) Two numerical simulations are conducted to validate the enhanced effectiveness of the proposed method. Sinusoidal signals are simulated with a small frequency shift compared to the FFT grids. In the presence of strong white Gaussian noise, RecAFSSC is superior to any other comparison technique in correcting information of frequency and phase for all possible normalized frequency error. RecAFSSC is also the most advantageous method for correcting the phase information when Δk∈(0,0.3), but only slightly inferior to the ratio-based spectral correction method, using a rectangular window for correcting the phase information when Δk∈(0.3,0.5). However, as to the amplitude, the original RecRB, even without the active frequency shifting process, is of the highest correction accuracy. Therefore, we combine the advantages of RecRB and those of RecAFSSC to design a procedure for the proposed method.
(3) A case study on the structural health monitoring of BFG power plant is conducted. There are 10 historical records of vibration signals before the occurrence of an accident involving the centrifugal fan. This event was caused by a crack that developed in the impeller blade. These data were collected from sensors mounted on the bearing housing. In analyzing the data, classical statistical indicators failed to reveal crack features as no explicit evolution trends with respect to time are detected. After applying the proposed AFSSC, the harmonic information related to fundamental working frequency is extracted with high precision. A normalized health state indicator, measuring the energy weight of each harmonic tone, is constructed. The results show that the normalized energy weight of the 2× component, the second-order harmonic tone of the fundamental working frequency, increases as time passes. This indicator successfully indicates the ongoing development of an incipient crack in the fault blade of the machinery.

## Figures and Tables

**Table 1:** Abbreviations of the proposed method and those of four comparison methods.

Abbreviations	Contents
HanRB	Ratio-based spectral correction technique using Hanning window.
HanAFSSC	HanRB using active frequency shifting operations as a preprocessing step.
RecRB	Ratio-based spectral correction technique using rectangular window.
RecAFSSC	RecRB using active frequency shifting operations as a pre-processing step.
AFSSC	The proposed method.

**Table 2:** Comparisons of the four techniques when $f_{c}=20.01\mathrm{Hz}$.

Spectral Information		HanAFSSC	HanRB	RecAFSSC	RecRB
Frequency (Hz)	Mean	0.0228	0.0282	0.0149	0.0209
	Std.	0.0168	0.0207	0.0113	0.0149
Amplitude	Mean	0.0325	0.0312	0.0274	0.0252
	Std.	0.0246	0.0235	0.0209	0.0195
Phase (rad)	Mean	0.0789	0.0926	0.0614	0.0715
	Std.	0.0593	0.0690	0.0468	0.0528

**Table 3:** Comparisons of the four techniques when $f_{c}=250.01\mathrm{Hz}$.

Spectral Information		HanAFSSC	HanRB	RecAFSSC	RecRB
Frequency	Mean	0.0232	0.0304	0.0148	0.0210
	Std.	0.0161	0.0218	0.0114	0.0153
Amplitude	Mean	0.0324	0.0306	0.0278	0.0251
	Std.	0.0248	0.0232	0.0209	0.0188
Phase	Mean	0.0797	0.0990	0.0637	0.0709
	Std.	0.0555	0.0717	0.0543	0.0581

**Table 4:** Comparisons of the four techniques when $f_{c}=480.01\mathrm{Hz}$.

Spectral Information		HanAFSSC	HanRB	RecAFSSC	RecRB
Frequency	Mean	0.0262	0.0302	0.0154	0.0284
	Std.	0.0173	0.0238	0.0113	0.0193
Amplitude	Mean	0.0325	0.0305	0.0280	0.0259
	Std.	0.0234	0.0221	0.0204	0.0185
Phase	Mean	0.0829	0.1025	0.0648	0.0926
	Std.	0.0622	0.0791	0.0488	0.0642

**Table 5:** Information of the 10 historical vibration tests (date, rotation speed, units).

Test Number	Interval (Days)	Sensors on Bearing Shell 2#			Sensors on Bearing Shell 3#			Remarks
		Axial (mm/s)	Horizon (mm/s)	Vertical (GE)	Axial (mm/s)	Horizon (mm/s)	Vertical (GE)	
1	-	L	L	L	L	L	L	Low speed operations (1-The initial test’)
2	99	L	L	L	L	L	L	
3	62	L	L	L	L	L	L	
4	35	L	L	L	L	L	L	
5	54	L	L	L	L	L	L	
6	29	H	H	H	H	H	H	High speed operations
7	5	H	H	H	H	H	H	
8	30	L	L	L	L	L	L	Low speed operations
9	29	L	L	L	L	L	L	
10	27	L	L	L	L	L	L	

**Table 6:** Corrected information of frequency, amplitude, and phase using the proposed AFSSC.

Test Number	Fundamental Frequency		Fundamental Frequency ×2		Fundamental Frequency ×3	
	Amplitude (mm·s^−1^)	Phase (°)	Amplitude (mm·s^−1^)	Phase (°)	Amplitude (mm·s^−1^)	Phase (°)
1	1.316	136.065	0.269	−33.388	0.199	125.074
2	1.844	−33.603	0.187	−71.140	0.195	−4.870
3	2.180	158.022	0.112	−63.192	0.181	−150.955
4	2.670	−173.618	0.151	−6.510	0.282	20.693
5	2.252	47.781	0.185	104.608	0.235	−110.818
6	9.590	−43.643	1.558	−49.710	0.900	62.944
7	9.372	−63.354	1.460	−101.821	0.706	−26.358
8	2.110	−45.932	0.752	153.325	0.357	−31.131
9	1.884	63.890	1.027	6.055	0.440	−50.505
10	1.795	−162.645	1.171	−91.907	0.361	1.491
